# Strategies for harm reduction in Latin America: the example of tobacco

**DOI:** 10.3389/fpubh.2025.1716852

**Published:** 2025-12-01

**Authors:** Enrique Teran

**Affiliations:** Colegio de Ciencias de la Salud, Universidad San Francisco de Quito USFQ, Quito, Ecuador

**Keywords:** tobacco and tobacco product, tobacco harm reduction, nicotine, Latin America, regulation and policy

## Abstract

Tobacco consumption continues to impose a profound public health and economic burden across Latin America, disproportionately affecting men, adolescents, and low-income populations. Despite progress in some countries through implementation of the World Health Organization (WHO) Framework Convention on Tobacco Control (FCTC), significant gaps remain due to weak regulatory frameworks, limited enforcement capacity, and persistent interference from the tobacco industry. Against this backdrop, tobacco harm reduction (THR), the substitution of combustible products with non-combustible or lower-exposure alternatives such as nicotine replacement therapies, electronic cigarettes, and heated tobacco products, offers a potentially valuable but underutilized strategy. Rather than a systematic review, this work offers a narrative, opinion-based synthesis of policy and evidence sources published between 2015 and 2024. While the WHO currently does not endorse electronic cigarettes or heated-tobacco products as cessation tools, the guiding principles of the WHO FCTC: proportional risk assessment, transparency, and surveillance, provide a conceptual basis for evaluating all nicotine-delivery systems under strict regulation. Latin-American governments should prioritize access to approved nicotine-replacement therapies and cessation services, while considering time-bounded, independent evaluation of non-combustible products within WHO FCTC guardrails where these are already present in the market. This perspective aims to inform balanced, evidence-based debate rather than advocate adoption of any specific product or policy.

## Introduction

Despite global progress in reducing smoking rates, tobacco consumption in Latin America remains a major public health challenge, particularly among men, adolescents, and vulnerable populations such as low-income groups and indigenous communities. The World Health Organization (WHO) estimates that tobacco kills more than eight million people annually worldwide, with a substantial proportion of these deaths occurring in low- and middle-income countries (1). While smoking prevalence has decreased in countries like Brazil and Uruguay, others, notably Bolivia and Paraguay, continue to report high use, especially among men and in rural settings (2). Smoking among adolescents is also concerning; for instance, surveys in Argentina indicate experimentation at early ages (3). Tobacco-related diseases remain leading causes of preventable death, lung cancer, chronic obstructive pulmonary disease (COPD), and cardiovascular disease, and impose significant economic costs (4, 5). Cultural and socioeconomic factors shape tobacco consumption patterns in the region. In Cuba and the Dominican Republic, tobacco has symbolic and economic relevance, complicating transitions away from combustible products (6). In Bolivia and Peru, lower-income populations face structural barriers to cessation services (3). Social dynamics, peer influence, normalized use in certain settings, and targeted marketing amplify uptake, particularly among youth (7).

In many Latin American societies, there is also a social component related to tobacco consumption, often perceived as a status marker or social activity. Young adults are particularly susceptible to peer pressure and social environments where smoking is normalized. Moreover, tobacco companies continue targeting these groups through indirect advertising, especially on social media and at musical events, despite the ban on traditional tobacco advertisements in many countries (7).

Implementation of FCTC measures has been uneven. Brazil’s comprehensive policies, such as plain packaging, graphic health warnings, taxation, and media campaigns, coincided with large declines in smoking since 1989 (8). Elsewhere, progress has lagged. Some countries have struggled even with basic smoke-free laws or sales restrictions to minors (9, 10). Enforcement gaps are compounded by illicit trade and the political influence of the tobacco industry (11, 12). Several Latin American countries are also significant tobacco producers; transitions must therefore account for rural livelihoods and trade dynamics (12).

Within this context, tobacco harm reduction (THR) has emerged internationally as a complementary approach to traditional tobacco control. THR emphasizes substituting combustible products with lower-exposure alternatives for adults who are unable or unwilling to quit. Nevertheless, adoption in Latin America has been slow due to regulatory bans, skepticism about long-term safety, and concerns about youth uptake.

This perspective paper is based on a narrative and non-systematic review of literature and policy documents published between 2015 and 2024. Sources were identified through PubMed, Scopus, WHO IRIS, and PAHO repositories, complemented by national government reports and public health statements relevant to Latin America. Because the objective was interpretative and policy-oriented rather than quantitative synthesis, inclusion was purposive, focusing on regional regulatory discussions and health-policy implications.

## Tobacco control and tobacco harm reduction (THR) approach in Latin America

Tobacco use remains heterogeneous across the region, with high prevalence pockets and persistent disparities by income, geography, and age (2–5). The tobacco industry’s influence and illicit trade complicate the policy environment (11, 12). Countries with comprehensive FCTC implementation (e.g., Brazil, Uruguay) have observed sustained declines in smoking (8), whereas others report slower progress (9, 10). Institutional capacity constraints, decentralized enforcement, and competing economic priorities contribute to uneven compliance (10–12). These realities frame the debate on whether and how THR could complement existing measures.

THR seeks to reduce exposure to toxicants by encouraging adult smokers who cannot or do not wish to quit to switch from combustible tobacco products (e.g., cigarettes) to lower-exposure alternatives such as nicotine replacement therapies (NRTs), electronic cigarettes (e-cigarettes), or heated tobacco products (HTPs). Combustion is the primary source of toxicants associated with smoking-related disease; nicotine, while addictive and not risk-free, is not the principal driver of smoking-related carcinogenesis (13). International evidence suggests that some non-combustible products can reduce exposure to harmful constituents compared with cigarettes and may support cessation for some users (14–16). However, the evidence is heterogeneous, and long-term health effects, especially cardiovascular outcomes and patterns of dependence, remain under investigation (17–24).

Several Latin American countries have adopted precautionary approaches to e-cigarettes and HTPs, ranging from strict regulation to outright bans, citing concerns over youth initiation, dual use, marketing practices, and uncertain long-term effects (13, 14, 17, 25–27). PAHO and WHO statements emphasize regulatory caution and comprehensive tobacco control integration (13, 17, 26). In this landscape, THR remains controversial and underdeveloped, despite potential benefits for subsets of adult smokers.

Reports from other regions describe reductions in biomarkers of exposure and, in some settings, declines in cigarette sales that temporarily coincide with the introduction of HTPs or wider e-cigarette uptake (14, 17, 19). For example, in Japan, cigarette sales declined substantially between 2016 and 2020, a trend that coincided with the introduction of HTPs and with other anti-smoking measures (14). These are associative observations within multifactorial contexts and do not establish causality; effects likely reflect combined influences of policy, pricing, enforcement, and cultural change.

## Recommendations for implementing effective harm reduction policies in Latin America

The implementation of tobacco harm reduction (THR) policies in Latin America presents unique challenges and opportunities. Below are key recommendations for policymakers and public health organizations to effectively promote THR while safeguarding public health and addressing concerns such as youth access and nicotine addiction.

### Development of a robust regulatory framework

Governments should enact risk-proportionate regulations ensuring product quality, age verification, ingredient disclosure, appropriate warnings, and marketing restrictions, while preventing the creation of unregulated markets. Regulatory clarity is preferable to prohibition-driven informality and illicit trade (12, 26).

### Public education and awareness

Public understanding of relative risks is limited. Communication should be balanced: acknowledging reduced exposure from non-combustible products compared with cigarettes, while clearly stating uncertainties about long-term health effects and the addictive nature of nicotine (15, 16, 19, 21–24, 28, 29). Education should avoid inadvertently promoting use among youth or non-smokers.

### Integration of THR into national tobacco control strategies

Many Latin American countries have successfully implemented the WHO Framework Convention on Tobacco Control (FCTC), which focuses on traditional tobacco control measures such as smoking bans, taxes, and public education (13). However, these measures alone may not be sufficient to address the diverse challenges posed by tobacco consumption in the region. THR should be integrated as a complementary component within FCTC-aligned strategies. Cessation remains the primary goal; for adults who cannot or will not quit, carefully regulated non-combustible options may offer risk-proportionate alternatives alongside clinical support (13, 15, 16, 23, 24, 33, 35).

### Equitable access to THR products

Given higher smoking prevalence among low-income and rural groups, policies should address access barriers to evidence-based cessation (NRT, counseling) and consider differential taxation, higher for combustibles, lower for certified lower-exposure products, while funding surveillance and enforcement (4, 5, 13, 36).

### Regulatory models applicable to Latin America

- *United Kingdom – risk-proportionate oversight*. Pre-market notification, toxicology data, quality controls, and strict marketing limits have been combined with cessation-oriented messaging and differential taxation (16).
- *Japan – fiscal and commercial restrictions*. HTPs are permitted under fiscal and product controls; nicotine e-liquids face distinct constraints. Products are taxed separately and carry warnings within the Tobacco Business Act framework (17).
- *Uruguay and Chile – incremental adaptation*. Within comprehensive tobacco laws, these jurisdictions have explored (or proposed) differentiated risk taxation, licensing, flavor controls, and surveillance of nicotine products in alignment with FCTC principles (26). A recent regional perspective underscores ongoing regulatory fragmentation and the need for evidence-informed coordination (30).

## Debunking myths about harm reduction in Latin America

Systematic and umbrella reviews highlight both potential benefits and unresolved risks, including cardiovascular effects and dependence, particularly among dual users (21–24). Evidence remains mixed on population-level cessation impact; some randomized and real-world studies report benefit for certain smokers, while others note persistent dual use (15, 23, 24).

Nicotine is a potent agonist of nicotinic acetylcholine receptors in central and peripheral pathways. Activation increases catecholamine release, producing transient rises in heart rate and blood pressure; chronic exposure may contribute to endothelial dysfunction and increased arterial stiffness, all those effects appear smaller in magnitude than with cigarette smoke but remain clinically relevant (21, 22). Nicotine engages mesolimbic dopaminergic pathways, reinforcing dependence via neuroadaptation. Systematic reviews indicate that e-cigarette or HTP users may develop sustained dependence, especially dual use (23). Withdrawal symptoms (irritability, anxiety, craving) underscore the need for clinical support even for users of reduced-exposure products.

Public discourse has sometimes conflated EVALI (E-cigarette, or vaping, product use-associated lung Injury) outbreaks, largely linked to illicit tetrahydro cannabinoid products containing vitamin E acetate, with regulated nicotine e-liquids, fueling confusion (28). Balanced communication should differentiate product categories, supply chains, and regulatory status.

Here, it was integrated evidence primarily from observational, ecological, and policy-level sources; accordingly, relationships described are correlational, not causal. Randomized or quasi-experimental data on THR outcomes in Latin America remain limited. Shifts in prevalence and health indicators often reflect concurrent changes in taxation, enforcement, social norms, and economic conditions. Recognizing these constraints prevents overstating the influence of any single intervention or product category.

Consistent with WHO’s precautionary position, e-cigarettes and heated-tobacco products are not currently recommended as cessation aids (4, 31, 36); therefore, any evaluation of such products must remain secondary to proven measures such as taxation, smoke-free environments, and accessible NRT-based cessation services. At the same time, the FCTC’s guiding principles: risk differentiation, proportional regulation, and protection of future generations, allow countries to assess exposure-reduction claims through independent scientific review rather than policy neglect (32).

Beyond causal limits, a balanced THR evaluation must consider the pharmacological burden of nicotine. Although nicotine is not the principal carcinogen in smoking, its sympathomimetic and pro-atherogenic pathways (e.g., endothelial dysfunction, oxidative stress, platelet activation) merit clinical and regulatory attention (21, 22). Dependence risks, especially among youth and dual users, require robust prevention, cessation support, and post-marketing surveillance (24). Within these guardrails, risk-proportionate regulation, aligned with WHO guidance, can accommodate the needs of adult smokers without compromising public health priorities.

Latin America faces persistent tobacco-related burdens amid regulatory heterogeneity and industry pressures (34). Harm reduction, integrated within comprehensive tobacco control and aligned to WHO recommendations, offers a pragmatic adjunct for adult smokers who cannot or do not quit, if access is risk-proportionate, youth protection is stringent, and long-term surveillance is continuous. By adopting evidence-informed policies tailored to regional realities, the region can reduce exposure and disease while safeguarding equity and public trust.

## Conclusion

Latin America faces persistent tobacco-related morbidity amid economic and regulatory diversity. Within the WHO–FCTC framework, the region’s priority remains to expand cessation coverage, enforce taxation and advertising bans, and safeguard youth.

Where non-combustible nicotine products are already in circulation, governments may consider conducting tightly regulated, evidence-based pilot evaluations, always subordinate to cessation priorities, guided by transparency, and insulated from tobacco industry influence. The concept of proportional risk, initially articulated by the Royal College of Physicians (15), may serve as a scientific framework for comparing toxicant exposure without implying safety or WHO endorsement.

This balanced interpretation situates harm-reduction debates inside, not outside, WHO’s comprehensive approach, emphasizing that proportional regulation and ethical oversight are complementary instruments for achieving the tobacco-free-generation goal.

## Data Availability

The original contributions presented in the study are included in the article/supplementary material, further inquiries can be directed to the corresponding author.
